# Supplementary material for: Loss of SRSF2 triggers hepatic progenitor cell activation and tumor development in mice
Source: Commun Biol. 2020 May 5;3:210. doi: 10.1038/s42003-020-0893-5 (PMC7200752; doi:10.1038/s42003-020-0893-5)
Supplement: Supplementary file 1 — Supplementary Information [file 42003_2020_893_MOESM1_ESM.pdf]

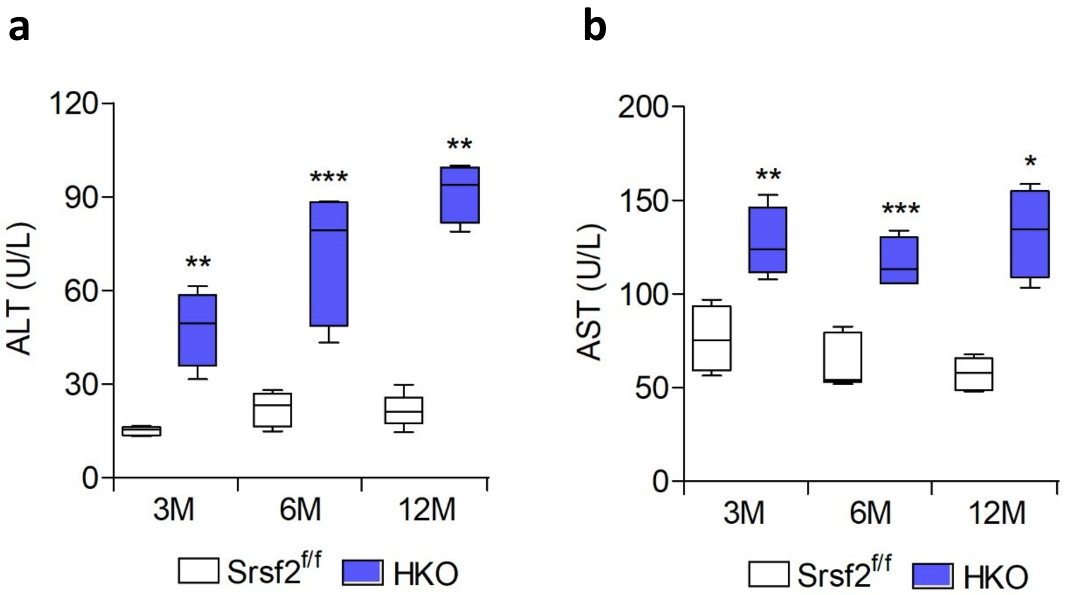

Supplementary Fig. 1 Chronic liver injury was present in the HKO mice. a , b Serum levels of alanine aminotransferase (ALT) (**a**) and aspartate aminotransferase (AST) (**b**) from Srsf2$^{f/f}$ and HKO mice at indicated ages (n=5).

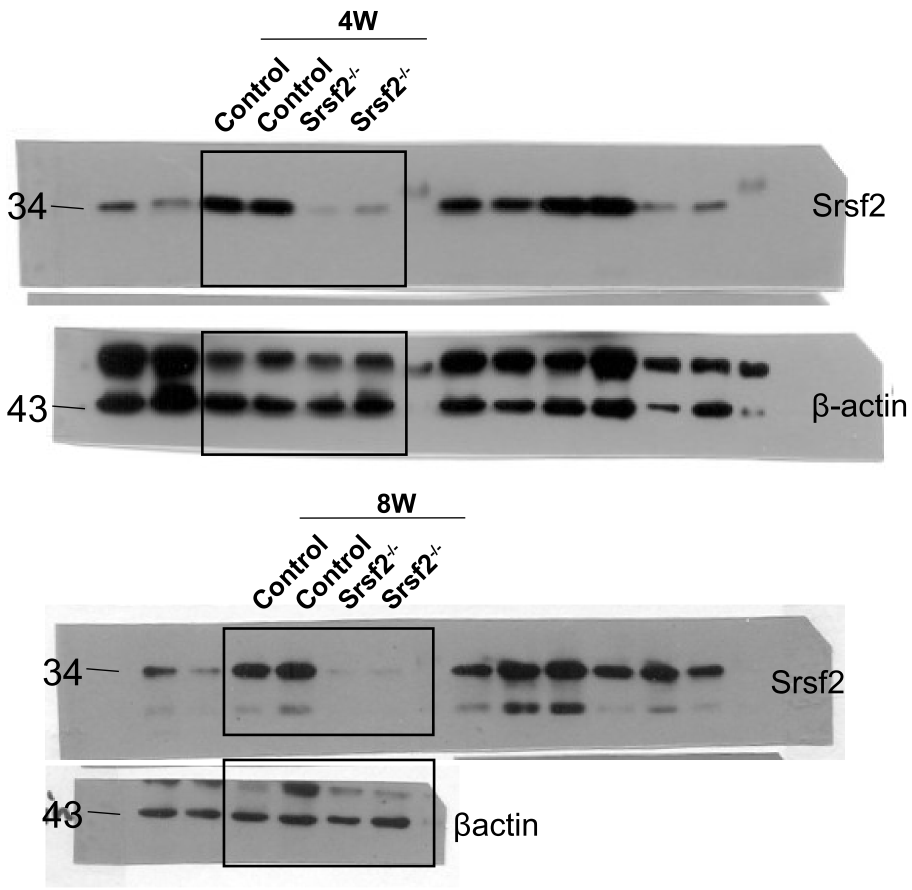

Supplementary Fig. 2 The original gel image of Fig. 4b.

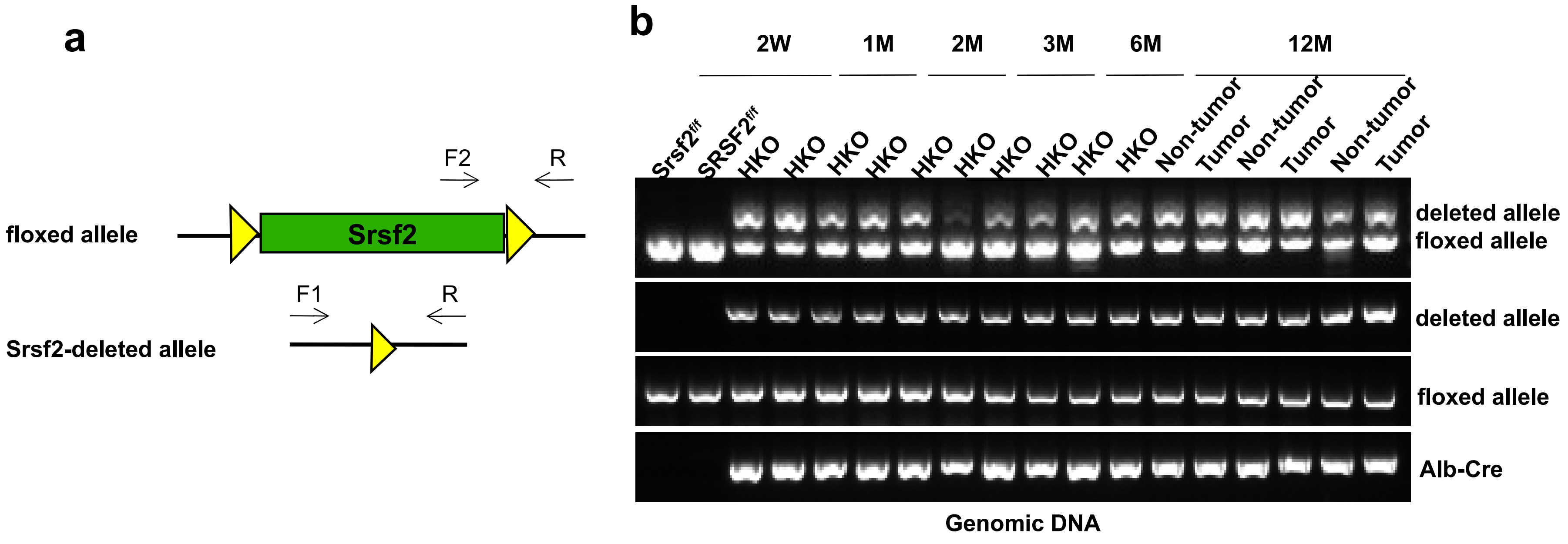

Supplementary Fig. 3 Deletion of *Srsf2* gene was examined in livers from mice at indicated ages by genomic DNA PCR. a Primer pairs (F2/R and F1/R) were designed for detection of floxed allele and deleted allele, respectively. **b** Genomic DNA was isolated from livers at indicated ages and used for genomic DNA PCR with combined F1/F2/R, F1/R or F2/R, respectively. In addition, Alb-cre was also analyzed by genomic DNA PCR.

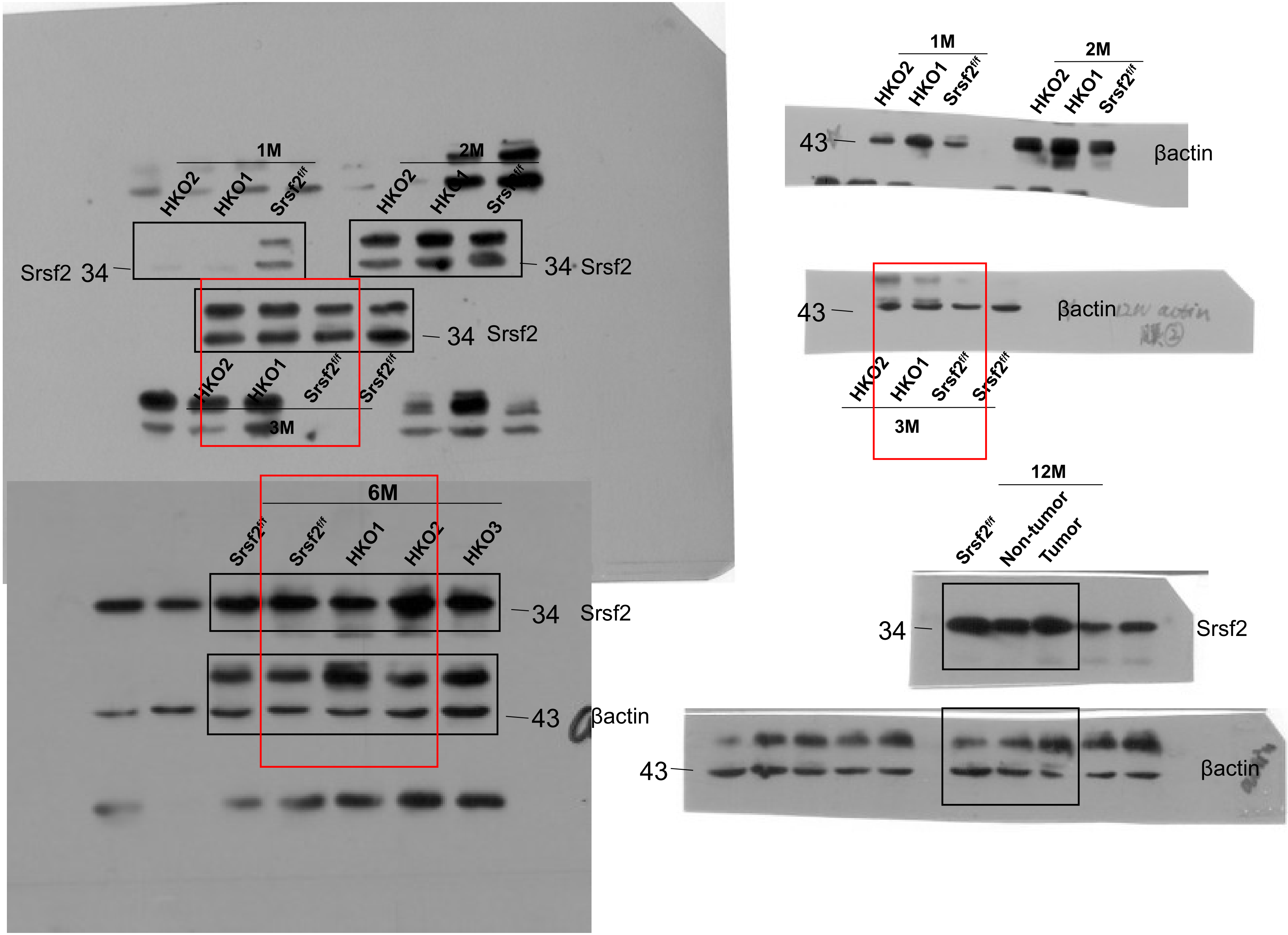

Supplementary Fig. 4 The original gel image of Fig. 6b.

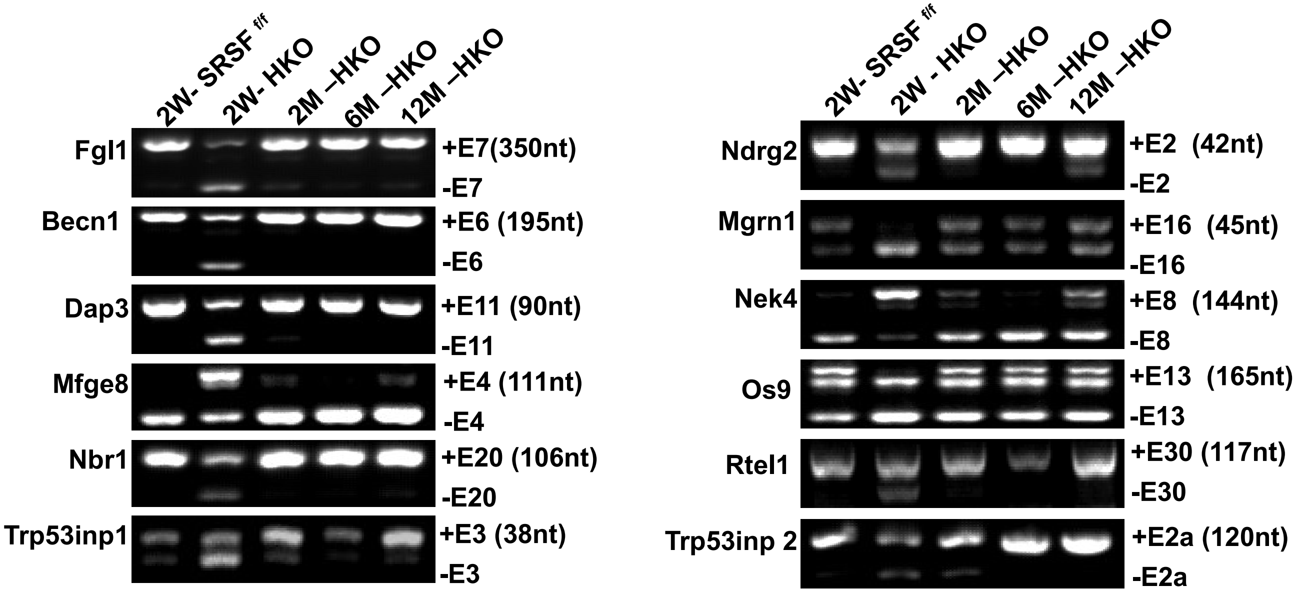

Supplementary Fig. 5 Alternative splicing events regulated by Srsf2 were not observed in livers of HKO mice at 2M, 6M or 12M. Total RNA was isolated from the liver of SRSF $^{f/f}$ mice at 2 weeks (2W), or from the liver of HKO mice at 2W, 2M, 6M or 12M. RT-PCR analysis was performed using primers for detecting alterative exon inclusion/skipping in the indicated genes. Note that alternative exons were regulated by Srsf2 in the 2W HKO liver, compared to 2W SRSF2$^{f/f}$ liver. However, those changes were not detected in the 2M, 6M or 12M HKO livers, which was consistent the fact that Srsf2 proteins were re-expressed in the 2M, 6M or 12M HKO livers.

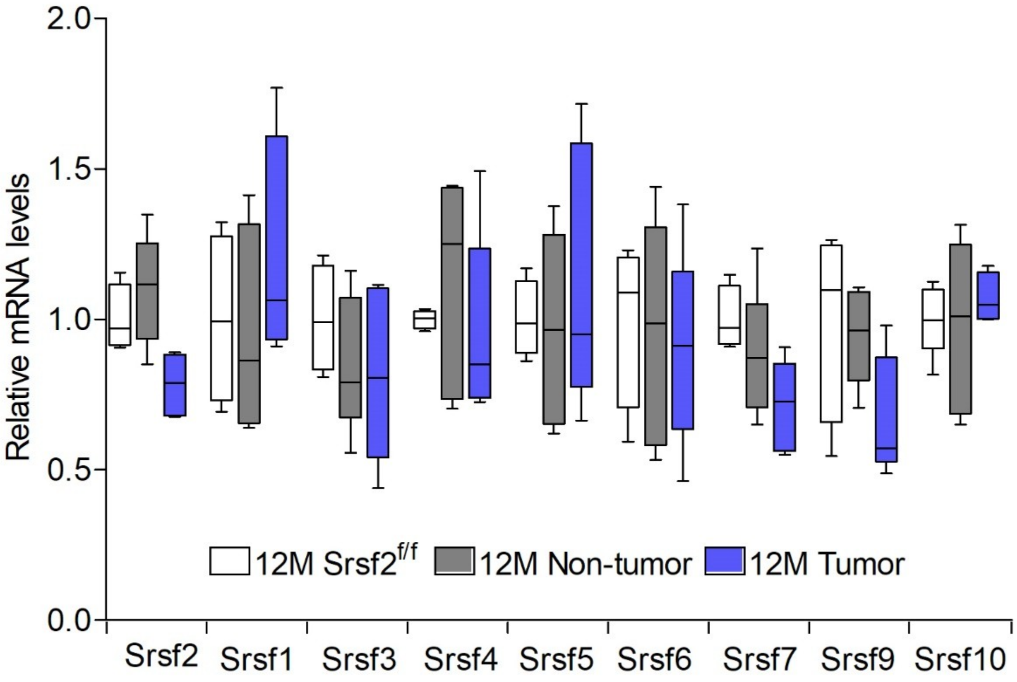

Supplementary Fig. 6 No compensatory changes were observed for other members of SR family in the HKO livers. qPCR analysis were performed using total RNA isolated from 12M control, 12M HKO non-tumor liver tissues or 12M HKO tumor tissues.

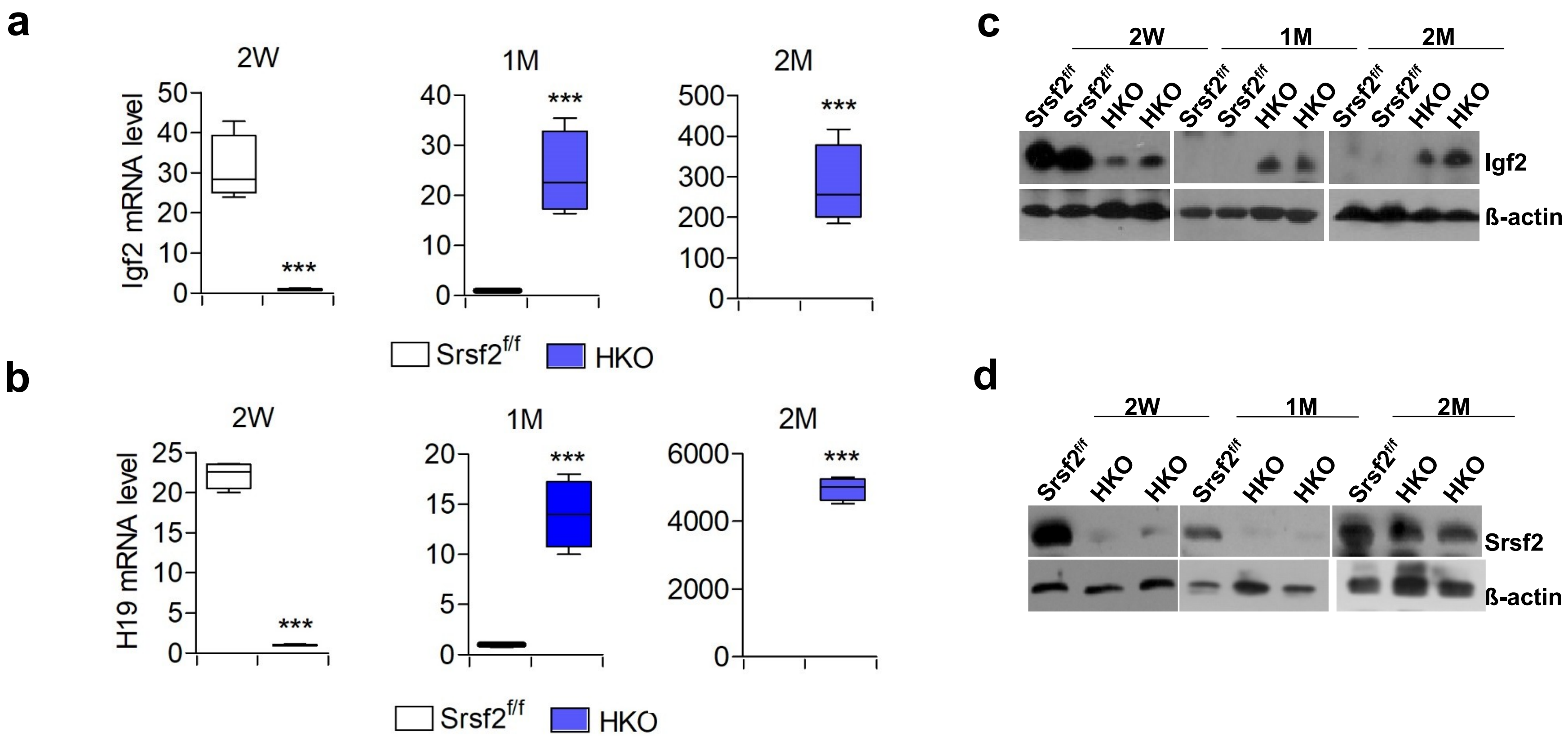

Supplementary Fig. 7 Liver regeneration was accompanied by up-regulation of Igf2/H19 in HKO livers. a, b Total RNA was isolated from livers of Srsf2$^{f/f}$ or HKO mice at 2W, 1M or 2M. qPCR analysis were performed to detect the Igf2 or H19 mRNA level. Results showed that both Igf2 and H19 were decreased at 2W but up-regulated at 1M and 2M. **c** Western blot analysis showed similar changes of Igf2 protein as its mRNA levels. **d** Western blot analysis of Srsf2.

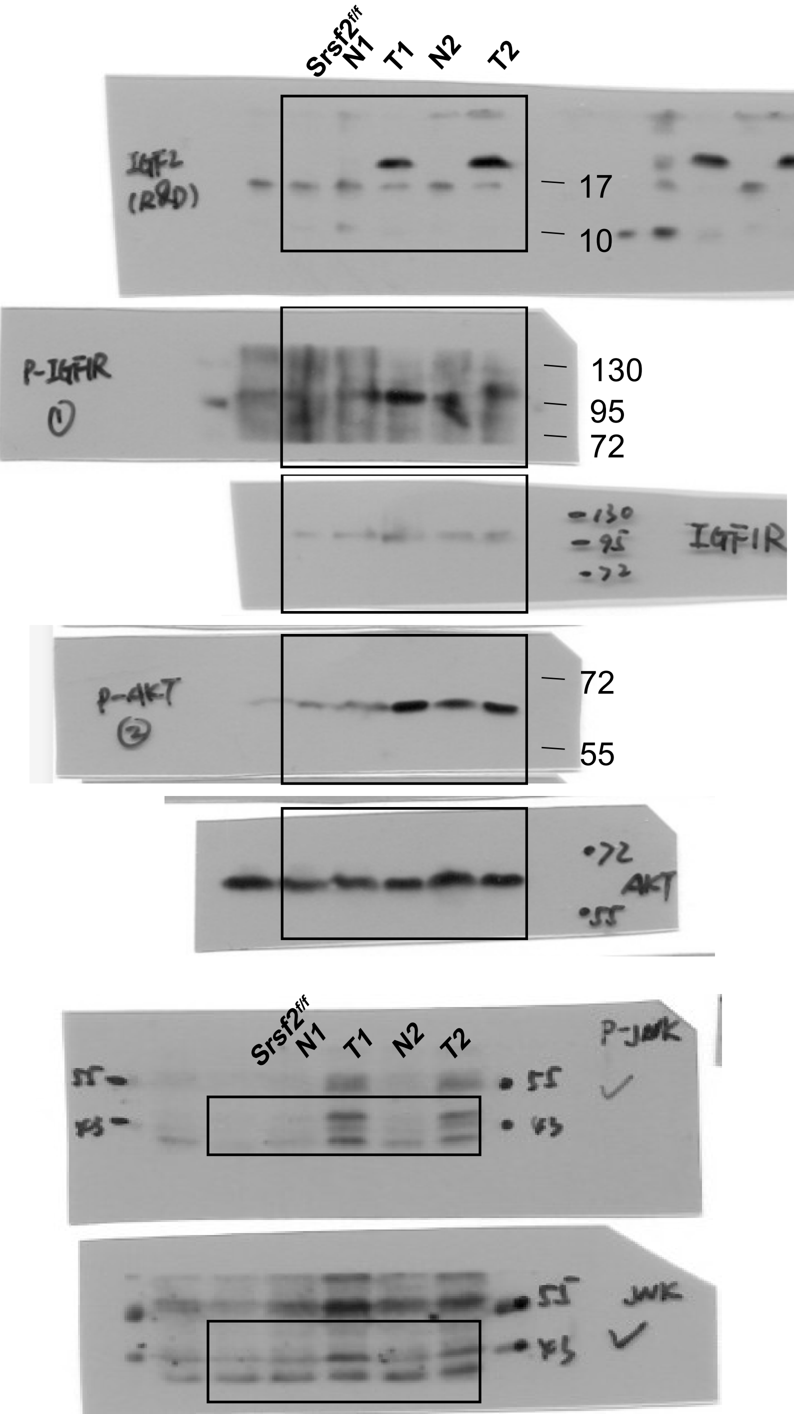

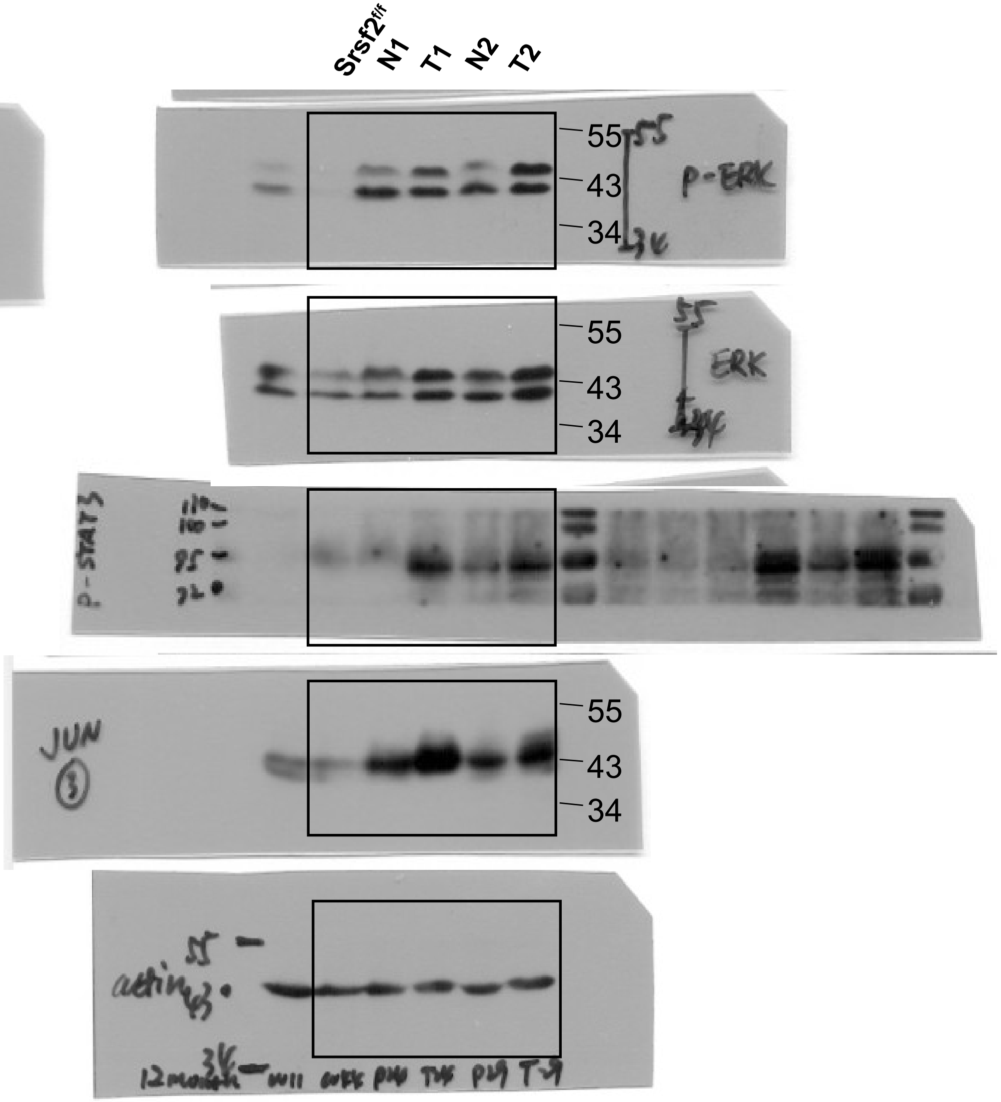

Supplementary Fig. 8 The original gel image of Fig. 7c.

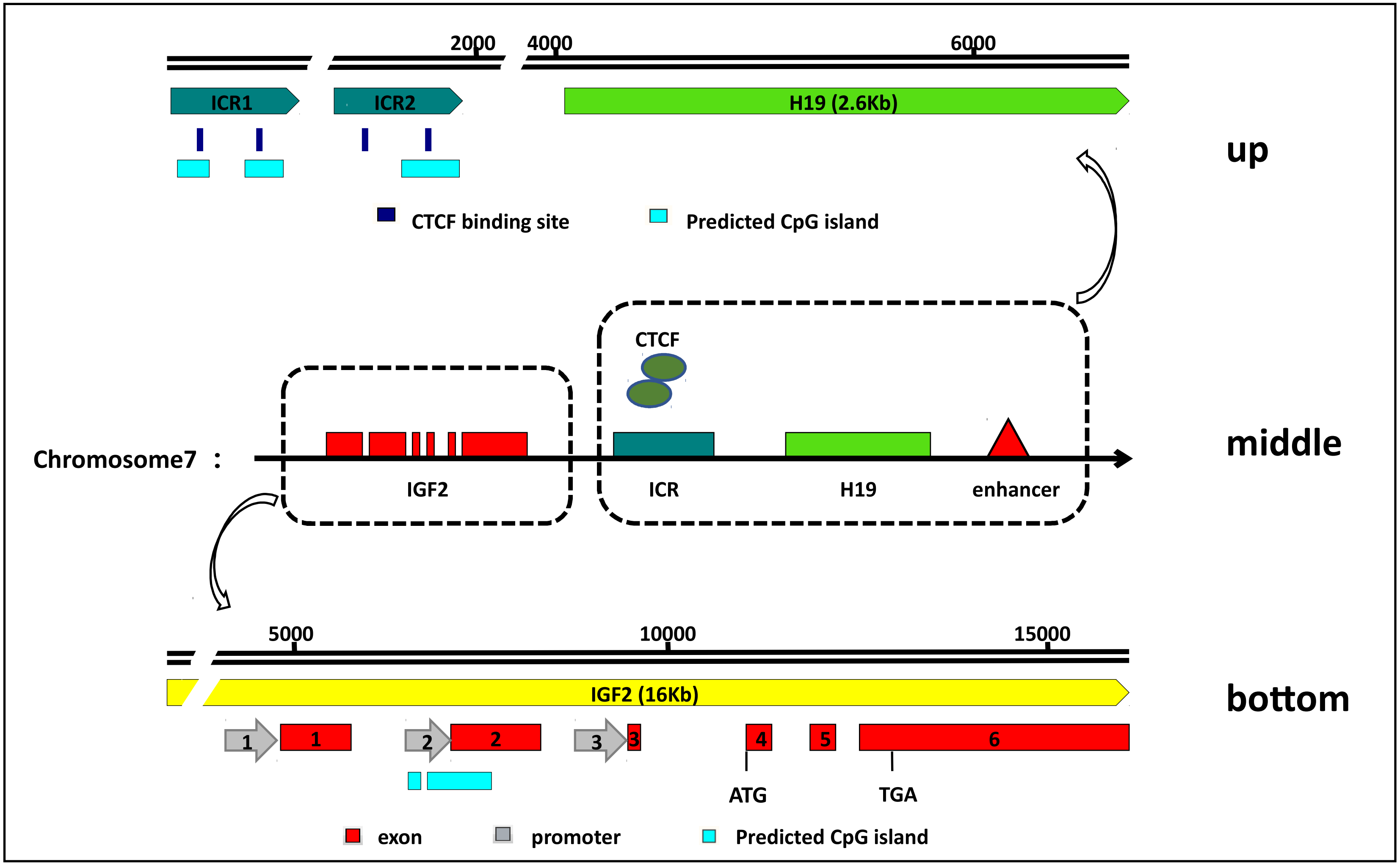

Supplementary Fig. 9 Structure of *Igf2/H19* locus on the chromosome in mice. The Igf2 was clustered with H19 on the chromosome 7 in mice controlled by an imprinting control region (ICR) in the 5'-flank of H19 and by a shared enhancer downstream of H19 **(middle)**.The ICR has two regions-ICR1 and ICR2, both of them contains two binding sites for an insulator protein-CCCTC binding factor (CTCF) **(up)**. Igf2 gene comprises four promoters (P0-P3, P1-P3 were shown in the picture) and 6 exons. Exons 4-6 encoded the entire pre-Igf2 protein **(down)**.

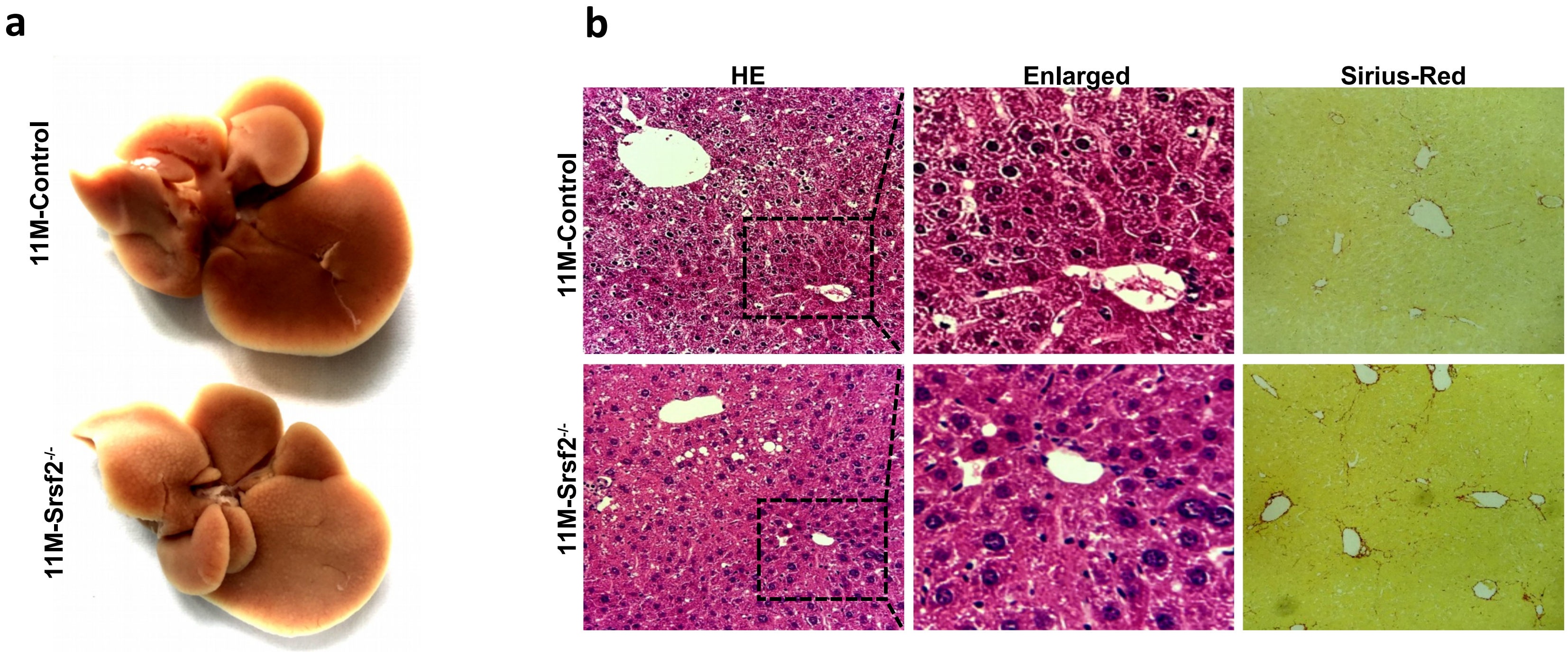

Supplementary Fig. 10 Tumors were not developed in livers of Srsf2$^{-/-}$ mice after 11 months of PolyI:C injection. **a** Representative photographs of livers from control and Srsf2$^{-/-}$ mice after 11 months of polyI:C treatment. However, no tumors were observed in both livers. **b** HE and Sirius-Red staining of livers from control and Srsf2$^{-/-}$ mice described in (**a**). Boxes were selected for magnification. Results showed that the Srsf2$^{-/-}$ livers displayed increased anisokaryosis and considerably high levels of fibrosis compared to controls.
