# Supplementary material for: Loss of SRSF2 triggers hepatic progenitor cell activation and tumor development in mice
Source: Commun Biol. 2020 May 5;3:210. doi: 10.1038/s42003-020-0893-5 (PMC7200752; doi:10.1038/s42003-020-0893-5)
Supplement: Supplementary file 7 — Description of Additional Supplementary Files [file 42003_2020_893_MOESM7_ESM.pdf]

Description for Supplementary Data as follows:

Supplementary Data 1: source data for Fig. 1

Supplementary Data 2: lists of antibodies and primer sequences for Fig. 1-7

Supplementary Data 3: source data for Fig. 2, 3

Supplementary Data 4: source data for Fig. 6

Supplementary Data 5: source data for Fig. 7
