# Supplementary material for: Loss of SRSF2 triggers hepatic progenitor cell activation and tumor development in mice
Source: Commun Biol. 2020 May 5;3:210. doi: 10.1038/s42003-020-0893-5 (PMC7200752; doi:10.1038/s42003-020-0893-5)
Supplement: Supplementary file 8 — Reporting Summary [file 42003_2020_893_MOESM8_ESM.pdf]

nature research

Corresponding author(s): Wenwu Wu and Ying Feng

Last updated by author(s): Feb 26, 2020

Reporting Summary

Nature Research wishes to improve the reproducibility of the work that we publish. This form provides structure for consistency and transparency in reporting. For further information on Nature Research policies, see Authors & Referees and the Editorial Policy Checklist.

Statistics

For all statistical analyses, confirm that the following items are present in the figure legend, table legend, main text, or Methods section.

n/a	Confirmed	
☐	☒	The exact sample size (*n*) for each experimental group/condition, given as a discrete number and unit of measurement
☐	☒	A statement on whether measurements were taken from distinct samples or whether the same sample was measured repeatedly
☐	☒	The statistical test(s) used AND whether they are one- or two-sided<br>*Only common tests should be described solely by name; describe more complex techniques in the Methods section.*
☒	☐	A description of all covariates tested
☒	☐	A description of any assumptions or corrections, such as tests of normality and adjustment for multiple comparisons
☐	☒	A full description of the statistical parameters including central tendency (e.g. means) or other basic estimates (e.g. regression coefficient) AND variation (e.g. standard deviation) or associated estimates of uncertainty (e.g. confidence intervals)
☐	☒	For null hypothesis testing, the test statistic (e.g. *F*, *t*, *r*) with confidence intervals, effect sizes, degrees of freedom and *P* value noted<br>*Give P values as exact values whenever suitable.*
☒	☐	For Bayesian analysis, information on the choice of priors and Markov chain Monte Carlo settings
☒	☐	For hierarchical and complex designs, identification of the appropriate level for tests and full reporting of outcomes
☒	☐	Estimates of effect sizes (e.g. Cohen's *d*, Pearson's *r*), indicating how they were calculated

Our web collection on statistics for biologists contains articles on many of the points above.

Software and code

Policy information about availability of computer code

Data collection	Microarray data of adult liver progenitor-enriched cells and depleted cells was from NCBI GEO (GSE29121) ；<br><br>Differentially expressed genes (DEGs) of PR-SET7-deficient mice liver were from a published paper (Spontaneous development of hepatocellular carcinoma with cancer stem cell properties in PR-SET7-deficient livers. The EMBO journal 34, 430-447 (2015)) .
Data analysis	Statistical tests in differential gene expression analysis of corresponding pairs (i.e. 2M HKO versus 2M Srsf2f/f, 12M non-tumorous section versus 12M Srsf2f/f, and 12M tumorous section versus non-tumorous section) were performed by EBSeq R package;<br><br>Differentially expressed probes of the microarray data (GSE29121) were obtained by using GEO2R tool;<br><br>GO and KEGG enrichment analysis were performed on the DEGs using TBtools.<br><br>The potential CpG island of imprinting control region (ICR) and IGF2 promoter were analysed by the online promoter analysis (http://www.urogene.org/cgi-bin/methprimer/methprimer.cgi) .

For manuscripts utilizing custom algorithms or software that are central to the research but not yet described in published literature, software must be made available to editors/reviewers. We strongly encourage code deposition in a community repository (e.g. GitHub). See the Nature Research guidelines for submitting code & software for further information.

Data

Policy information about availability of data

All manuscripts must include a data availability statement. This statement should provide the following information, where applicable:

- Accession codes, unique identifiers, or web links for publicly available datasets
- A list of figures that have associated raw data
- A description of any restrictions on data availability

The raw RNA-sequencing data had been deposited to Gene Expression Omnibus (GEO) with accession number GSE130745.

Field-specific reporting

Please select the one below that is the best fit for your research. If you are not sure, read the appropriate sections before making your selection.

☒ Life sciences ☐ Behavioural & social sciences ☐ Ecological, evolutionary & environmental sciences

For a reference copy of the document with all sections, see nature.com/documents/nr-reporting-summary-flat.pdf

Life sciences study design

All studies must disclose on these points even when the disclosure is negative.

Sample size	We used >5 independent samples for relevant analysis and delete the maximal and minimal results when making a box-and- whisker (e.g. qPCR, methylation analysis, ALT or AST examination, body weight or ratios of liver weight/spleen weight to body weight) .<br><br>When confirming the survival of HKO mice, we collected 136 HKO mice.<br><br>For each staining, we used at least 3 HKO livers for analysis for excluding individual difference.<br><br>For EdU assay, we used at least 3 Srsf2f/f and HKO mice for analysis.<br><br>All data presented as box-and-whisker refer to a mean value ± SEM of the total number of independent experiments. Statistical analysis was performed by Student's t-test at a significance level of $P<0.05$, $**P<0.01$,$***P<0.001$.
Data exclusions	The Oil-Red staining (6month and 12 month) and blood glucose detection (2month and 6month). There are no significance between control mice and HKO livers. So we can exclude the possibility of non-alcoholic fatty liver disease /NAFLD - induced HCC, and also exclude the direct glucose deficiency-caused death.
Replication	We used >5 independent samples for relevant analysis and delete the maximal and minimal results when making box-and-whisker.<br><br>When confirming the survival of HKO mice, we collected 136 HKO mice.
Randomization	For survival confirmation, body weight measurement, we obtained the results in batches without any exclusion.<br>For ALT or AST examination,qPCR, we collected >5 HKO mice at a given stage, all livers of these mice were succumbed to mRNA extraction without deliberate exclusion.<br>For western blot, we randomly selected 2 HKO or Srsf2-/- livers for analysis.
Blinding	Most of the investigations were blinded to group allocation.<br>Some were not, we select the considerable malignant livers for RNA-seq but this is rational. Because most of the phenotypes of HKO liver were similar, in order to accurately obtain the differentially expressed genes, the more malignant livers may be optional.

Reporting for specific materials, systems and methods

We require information from authors about some types of materials, experimental systems and methods used in many studies. Here, indicate whether each material, system or method listed is relevant to your study. If you are not sure if a list item applies to your research, read the appropriate section before selecting a response.

Materials & experimental systems

n/a	Involved in the study
☐	☒ Antibodies
☒	☐ Eukaryotic cell lines
☒	☐ Palaeontology
☐	☒ Animals and other organisms
☒	☐ Human research participants
☒	☐ Clinical data

Methods

n/a	Involved in the study
☒	☐ ChIP-seq
☒	☐ Flow cytometry
☒	☐ MRI-based neuroimaging

Antibodies

Antibodies used

Ki67 BD (550609)
AFP proteintech (14550-1-AP)
GS Santa Cruz (sc74430)
P62 Sigma (P0067)
CD45 Abcam (ab10558)
γH2AX Abways (CY6572)
P21 Abcam (ab109199)
Hnf4α CST (3113)
Hnf4α Abcam (ab41898)
A6 DHSB (A6 BCM-S)
CK19 DHSB (Trom-III)
CK19 Abcam (ab133496)
sox9 Mllipore (AB5535)
αSMA Abways (CY5027)
Laminin Abcam (ab11576)
FGF7 R&D (AF-251-NA)
F4/80 ebioscience (11-4801-85)
SRSF2 Novus (NBP2-47290)
βactin Santa Cruz (sc47778)
IGF2 R&D (AF-792)
pIGF1R CST (3024)
IGF1R CST (9750)
pAKT CST (4060)
AKT CST (4691)
pJNK CST (9251)
JNK CST (9252)
pERK1/2 CST (9101)
ERK1/2 CST (4695)
pSTAT3 CST (9145)
JUN CST (9165)

Validation

All the primary antibodies are available in mouse. (Note: IF represents Immunofluorescence; P represents Paraffin; F represents Frozen; WB represents Western Blot; IHC represents Immunohistochemistry)
Ki67 IF-P(1:100)
AFP IHC(1:50)
GS IHC(1:50)
P62 IF-P/F(1:100)
CD45 IF-F(1:100)
γH2AX IF-F(1:200)
P21 IF-P(1:100)
Hnf4α IF-P/F(1:400)
Hnf4α IF-P(1:50)
A6 IF-F(1:50)
CK19 IF-P/F(1:100)
CK19 IF-F(1:500)
sox9 IF-P/F(1:100)
αSMA IF-F(1:100)
Laminin IF-F(1:100)
FGF7 IF-F(5-15 μg/mL)
F4/80 IF-F(1:100)
SRSF2 WB(1:5000)
βactin WB(1:1000)
IGF2 WB(1:1000)
pIGF1R WB(1:1000)
IGF1R WB(1:1000)
pAKT WB(1:1000)
AKT WB(1:1000)

pJNK WB(1:1000)
JNK WB(1:1000)
pERK1/2 WB(1:1000)
ERK1/2 WB(1:1000)
pSTAT3 WB(1:1000)
JUN WB(1:1000)

Animals and other organisms

Policy information about studies involving animals; ARRIVE guidelines recommended for reporting animal research

Laboratory animals	C57BL/6J, all the mice used for experimental immunostaining and EdU assay were regardless of sex. we mainly focus on the mice at age of 1-3 month, 6 month, 12 month.<br>The qPCR, blood examination mainly used the male mice.
Wild animals	Study didn't involve the wild animals
Field-collected samples	Study didn't involve the animals collected from the field.
Ethics oversight	All experiments were conducted in accordance with the guidelines of the Institutional Animal Care and Use Committee of the Institute for Nutritional Sciences, Shanghai Institute for Biological Sciences, Chinese Academy of Sciences.

Note that full information on the approval of the study protocol must also be provided in the manuscript.
